# RBM5/LUCA-15 — Tumour Suppression by Control of Apoptosis and the Cell Cycle?

**DOI:** 10.1100/tsw.2002.859

**Published:** 2002-07-04

**Authors:** Mirna Mourtada-Maarabouni, Gwyn T. Williams

**Affiliations:** School of Life Sciences, Keele University, Keele, Staffs, ST5 5BG, UK

**Keywords:** T-lymphocytes, LUCA-15, RNA-binding proteins, cell death, cell proliferation

## Abstract

The candidate tumour suppressor gene, LUCA-15, maps to the lung cancer tumour suppressor locus 3p21.3. The LUCA-15 gene locus encodes at least four alternatively spliced transcripts, which have been shown to function as regulators of apoptosis, a fact that may have a major significance in tumour regulation. This review highlights evidence that implicates the LUCA-15 locus in the control of apoptosis and cell proliferation, and reports observations that significantly strengthen the case for tumour suppressor activity by this gene.

## INTRODUCTION

The molecular control of cancer is complex. Cancer genes are involved in the dysregulation of a whole range of normal cellular systemic processes including cell proliferation[1], cell-cell communication[2], DNA repair[3], chromosome stability[4], tumour invasion, motility and metastasis[5,6], apoptosis[7], and others. Despite impressive progress in recent years in understanding the molecular basis of cancer, many crucial genes remain to be identified. One or more such genes are located at the human chromosomal locus 3p21.3, which is strongly associated with lung cancer and many other cancers, including head and neck, renal, breast, and female genital tract[8,9]. More than 90% of freshly microdissected primary lung cancers experience loss of heterozygosity (LOH) of 3p21.3, and the fact that no single locus of this region appears to be consistently affected by LOH suggests that the region harbours more than one tumour suppressor genes (TSGs)[10,11]. The search for TSGs in this region has, therefore, been the focus of extensive investigations over the past 10 years, and many genes mapping to this region have recently been cloned[12,13,14].

One of the genes encoded at 3p21.3, LUCA-15 (Accession No. U73168), which was first isolated in an attempt to define the genes associated with a 370-kb overlapping lung cancer homozygous deletion region[12]. Timmer et al.[8] also reported the cloning of the same gene, naming it RBM5 (RNA-Binding Motif protein 5; Accession No. AAD04159)[8]. In this mini-review, the effects of LUCA-15 and its splice variants are discussed in the context of regulation of apoptosis and cell proliferation. Although there is limited literature on this aspect, the evidence available strongly suggests that LUCA-15 plays an important role in regulation of cell proliferation and apoptosis and thus elucidation of its mechanisms of action should advance our understanding both of apoptosis and of oncogenesis.

## LUCA-15: AN RNA-BINDING PROTEIN

Luca-15 was initially cloned as a putative TSG mapping to 3p21.3[8,12], and its primary sequence indicates a number of functional motifs. These include two RNA binding domains, two putative zinc-finger DNA binding motifs, two bipartite nuclear localisation signals and the recently identified G-patch domain (a conserved domain in eukaryotic RNA-processing proteins and type D retroviral polyproteins)[15], suggesting a role for LUCA-15 in RNA processing. The N-terminal fragment of human LUCA-15 containing the RNA binding domain[16] and an epitope-tagged LUCA-15 protein were found to interact preferentially with poly (G) RNA homopolymer *in vitro*[17]. In addition, the C-terminal region of LUCA-15 contains several domains including a glutamine rich domain (362–385), which is thought to serve as protein–protein interaction site in certain RNA- and DNA- binding proteins[17].

RBM5 and its immediately telomeric neighbour RBM6, also a candidate TSG, share 30% amino acid sequence identity, indicating that they are likely to be part of the same gene family and arose through gene duplication[7,8,16,19,20]. Interestingly, the RBM6 sequence is identical to the sequence of the NY-LU-12 gene (Accession No. AF04285), which was identified by screening a recombinant lung cancer cDNA expression library with autologous patient antisera[19] and to the Def-3 clone (Accession No. AF069517), a gene which is differentially expressed during myelopoiesis[8,16], suggesting that RBM6 might be involved in cancer development.

## REGULATION OF APOPTOSIS AND CELL PROLIFERATION BY LUCA-15 cDNA FAMILY

LUCA-15 is ubiquitously expressed in human tissues[16], but its function has not been established, although the fact that LUCA15 expression is high in the adult thymus and low in the foetal thymus indicates that its expression can be developmentally regulated[16]. Although the LUCA-15 gene has not been shown to have an increased mutation rate in cancers, a number of lines of evidence do suggest its involvement in apoptosis and in cell cycle regulation. LUCA-15 is reported to be downregulated both in RAS-transformed Rat-1 cells and in samples from breast cancer[17]. In addition, overexpression of LUCA-15 in human fibrosarcoma HT1080 cells suppressed cell growth[17]. Finally, LUCA-15 was one of the antigens identified by autologous antibody in patients with renal carcinoma[21].

The first piece of evidence that supports a possible role for LUCA-15 in apoptosis derives from the studies performed by Sutherland and colleagues[22]. Using an episomal retrieval functional expression cloning system[23], a 326bp bone marrow cDNA fragment (termed Je2; Accession No. AF107492), which strongly inhibits Fas-mediated apoptosis in Jurkat T-cells, was identified[22]. Je2 lies within an intron of the LUCA-15 gene (Accesssion No.U23946), in the

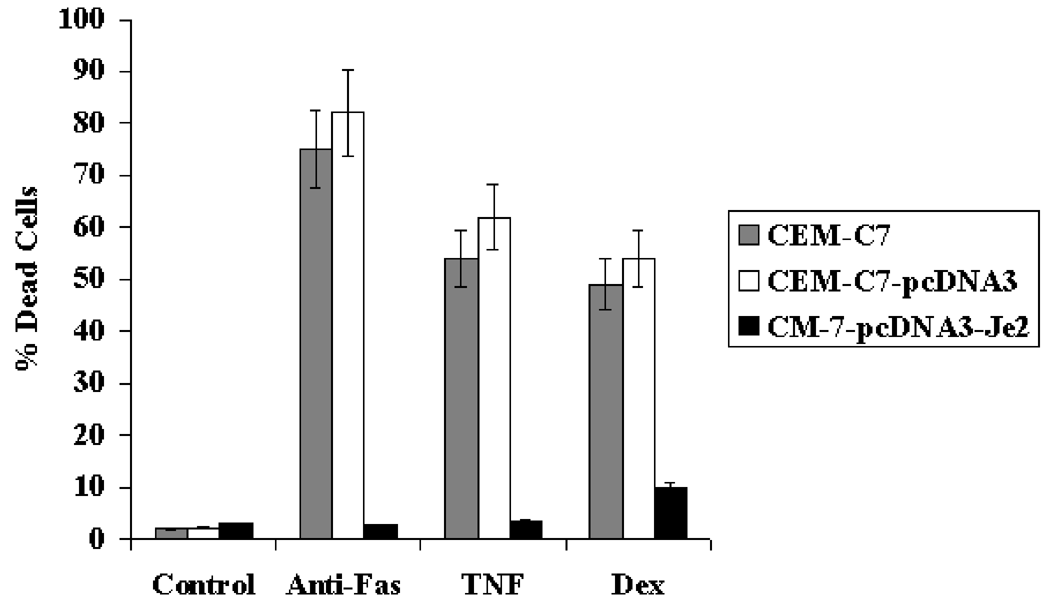

**FIGURE 1**. Je2 over-expression inhibits CD95-, TNFα-, and dexamethasone- mediated cell death. CEM-C7 cells were stably transfected with pcDNA3 vector alone or pcDNA3-Je2. Cells ($2 \times 10^5$ cells/ml) were incubated in the presence of 5 ng/ml anti-CD95, TNFα 50 ng/ml, and dexamethasone 10 μM for 48 h. The percentage of dead cell number was determined by nigrosin dye exclusion.

opposite orientation to LUCA-15 transcription[12]. Stable expression of Je2 in another human T-cell line CEM-C7 produced marked inhibition of Fas-mediated apoptosis and conferred protection from apoptosis induced by other stimuli (Fig. 1)[24]. Since Je2 is complementary to part of the LUCA-15 locus, it is likely to suppress apoptosis in the CEM-C7 cell line through a direct antisense effect on LUCA-15 RNA. Interestingly, the LUCA-15 polypeptides present are altered significantly in CEM-C7–Je2 stable clones [including downregulation of full-length LUCA-15 protein: Mourtada-Maarabouni and Williams, manuscript in preparation], indicating that Je2 might function to inhibit apoptosis by interfering with the processing of LUCA-15 transcripts. In addition stable expression of Je2 altered the expression of several genes[24] (Table 1), some of which have been previously reported to regulate apoptosis.

**TABLE 1**
**Fold Change and Entrez Definition of the Top 7 Differentially Regulated Genes in Je2 -Expressing Cells**

| Entrez Definition | Fold |
|---|---|
| **Genes downregulated** | |
| Caspase-10 | 5 |
| B-cell lymphoma protein 7B | 6 |
| Interferon- inducible protein | 15 |
| Transcription factor Stat5b | 17 |
| **Genes upregulated** | |
| B-cell translocation gene 2(BTG2) | 12 |
| Pim-1-oncogene | 7 |
| Checkpoint suppressor 1- mRNA | 5 |

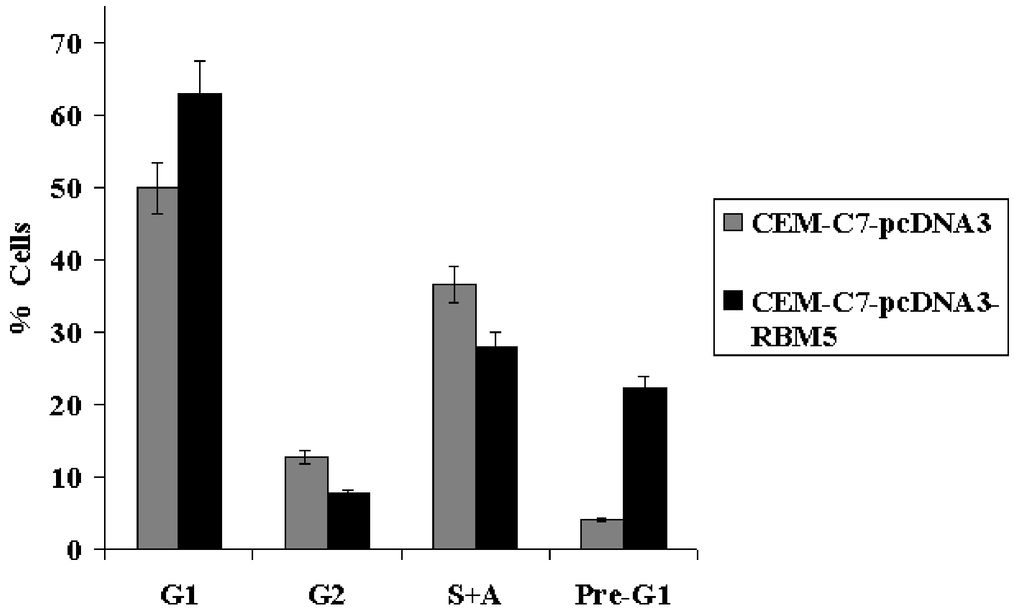

**FIGURE 2**. LUCA-15/RBM5 is capable of inducing cell cycle arrest in $G_1$-M and apoptosis. DNA content from CEM-C7/pcDNA3 and CEM-C7/pcDNA3-RBM5 was quantified by propidium iodide staining of fixed cells.

A second piece of evidence has also been obtained which demonstrates that LUCA-15 is a negative regulator of cell proliferation. Overexpression of the full-length LUCA-15/RBM5 in CEM-C7 and Jurkat T-cell lines suppressed cell proliferation both by inducing apoptosis and by producing a modest but significant accumulation of cells in $G_1$-M, suggesting that LUCA-15 may independently function to induce apoptosis (Fig. 2)[25; Mourtada-Maarabouni and Williams, submitted for publication]. In addition, Microarray analysis indicated that overexpression of LUCA-15/RBM5 altered the expression of the same genes affected by Je2 in the opposite direction[25], as would be expected if Je2 is indeed acting as an antisense sequence.

A third, and still more convincing, line of reasoning derives from the fact that splice variants of the LUCA-15 gene have been shown to function as regulators of apoptosis[22]. Both alternative RNA splicing and antisense transcription occur at the LUCA-15 gene locus[22]. LUCA-15 splice variants display antagonistic properties that include promotion of apoptosis and inhibition of proliferation by transcripts encoding full-length LUCA-15[22; Mourtada-Maarabouni and Williams; submitted for publication]. In direct contrast, LUCA-15Δ6, a variant lacking exon 6 and producing a truncated protein, is upregulated in some T-cell leukaemic lines and inhibits CD95-mediated apoptosis as well as accelerating proliferation[Mourtada-Maarabouni and Williams, submitted for publication].

Taken together, all of the foregoing evidence is consistent with the concept that LUCA-15 plays an important role in the regulation of cell proliferation and apoptosis. LUCA-15 belongs to a large family of related proteins that have diverse functions in the regulation of gene expression[26]. Members of this RNA-binding protein superfamily are involved in various aspects of RNA synthesis, alternate splicing, RNA editing, and metabolism[26]. In addition, LUCA-15 contains a glutamine rich domain, which is thought to serve as protein–protein interaction site. Thus, it is likely that LUCA-15 may regulate the expression of genes responsible for the control of the cell cycle and apoptosis by both transcriptional and post-transcriptional mechanisms. Identification of the RNA and proteins that interact with LUCA-15 should provide more information into its function.

## CONCLUSION

There are 12 genes, including p53, currently accepted as *bona fide* tumour suppressors, whose mutation or deletion is associated with tumourigenesis in human cancers[27]. The ability of LUCA-15 to inhibit the growth of transformed cells fulfils one of the criteria for a tumour suppressor. In addition, LUCA-15 is located at an important site on chromosome 3, a region highly susceptible to aberrant chromosomal rearrangement and deletions, suggesting the presence of TSG at this locus[7,8,9]. Any novel gene mapping to this region is therefore recognised as a putative TSG[7,8,9,10], and the simultaneous changes in proliferation and inhibition of apoptosis brought about by dysregulation of the LUCA-15 locus could therefore be of major significance in cancer development. Further studies are required to delineate the mechanism of functions of LUCA-15 and its splice variants in regulating apoptosis. This may clarify the function of LUCA-15 as a TSG and define its importance in oncogenesis.

## ACKNOWLEDGEMENT

This work was supported by the Wellcome Trust.

## References

1. Hunt, T. and Nasmyth, K. (1997) Cell multiplication. *Curr. Opin. Cell Biol.* **9,** 765–767.
2. Hirschi, K.K., Xu, C.E., Tsukamoto, T., and Sager, R. (1996) Gap junction genes Cx26 and Cx43 individually suppress the cancer phenotype of human mammary carcinoma cells and restore differentiation potential. *Cell Growth Differ.* **7**, 861–870.
3. Sancar, A. (1995) DNA repair in humans. *Annu. Rev. Genet.* **29**, 69–105.
4. Hanawalt, P.C. (1994) Transcription-coupled repair and human disease. *Science* **266**, 1957–1958.
5. Muller, B.M., Yu, Y.B., and Lang, W.E. (1995) Overexpression of plasminogen activator inhibitor 2 in human melanoma cells inhibits spontaneous metastasis in scid/scid mice. *Proc. Natl. Acad. Sci. U. S. A.* **92**, 205–209.
6. Stetler-Stevenson, W.G., Aznavoorian, S., and Liotta, L.A. (1993) Tumor cell interactions with the extracellular matrix during invasion and metastasis. *Annu. Rev. Cell Biol.* **9**, 541–573.
7. Kok, K., Naylor, S.L., and Buys, C.H. (1997) Deletion of the short arm of chromosome 3 in solid tumors and the search for suppressor genes. *Adv. Cancer Res.* **71**, 27–92.
8. Timmer, T., Terpstra, P., van den Berg, A., Veldhuis, P.M., Ter Elst, A., Voutsinas, G., Hulsbeek, M.M., Draaijers, T.G., Looman, M.W., Kok, K., Naylor, S.L., and Buys, C.H. (1999) A comparison of genomic structures and expression patterns of two closely related flanking genes in a critical lung cancer region at 3p21.3. *Eur. J. Hum. Genet.* **7**, 478–486.
9. Lerman, M.I. and Minna, J.D. (2000) The 630-kb lung cancer homozygous deletion region on human chromosome 3p21.3: identification and evaluation of the resident candidate tumor suppressor genes. *Cancer Res*. **60**, 6116–6133.
10. Daly, M.C., Xiang, R.H., Buchhagen, D., Hensel, C.H., Garcia, D.K., Killary, A.M., Minna, J.D., and Naylor, S.L. (1993) A homozygous deletion on chromosome 3 in a small cell lung cancer cell line correlates with a region of tumor suppressor activity. *Oncogene* **8**,1721–1729.
11. Kashuba, V.I., Szeles, A., Allikmets, R., Nilsson, A.S., Bergerheim, U.S., Modi, W., Grafodatsky, A., Dean, M., Stanbridge, E.J., Winberg, G., Klein, G., Zabarovsky, E.R., and Kisselev, L. (1995) A group of NotI jumping and linking clones cover 2.5 Mb in the 3p21-p22 region suspected to contain a tumor suppressor gene. *Cancer Genet. Cytogenet*. **81**,144–150.
12. Wei, M.H., Latif, F., Bader, S., Kashuba, V., Chen, J.Y., Duh, F.M., Sekido, Y., Lee, C.C., Geil, L., Kuzmin, I., Zabarovsky, E., Klein, G., Zbar, B., Minna, J.D., and Lerman, M.I. (1996) Construction of a 600-kilobase cosmid clone contig and generation of a transcriptional map surrounding the lung cancer tumor suppressor gene (TSG) locus on human chromosome 3p21.3: progress toward the isolation of a lung cancer TSG. *Cancer Res*. **56**, 1487–1492.

13. Imreh, S., Kost-Alimova, M., Kholodnyuk, I., Yang, Y.,Szeles, A., Kiss, H., Liu, F., Foster, K., Zabarovsky, E., Stanbridge, E., and Klein, G. (1997) Differential elimination of 3p and retention of 3q segments in human/mouse microcell hybrids during tumor growth. *Genes Chrom. Cancer*. **20**, 224–233.
14. Daigo, Y., Nishiwaki, T., Kawasoe, T., Tamari, M., Suchiya, E., and Nakamura. Y. (1996) Molecular cloning of a candidate tumor suppressor gene, DLC1, from chromosome 3p21.3. *Cancer Res*. **59**:1966–1972.
15. Aravind, L. and. Koonin, E.V. (1999) G-patch: a new conserved domain in eukaryotic RNA-processing proteins and type D retroviral polyproteins. *Trends Biochem. Sci.* **24**, 342–344
16. Drabkin, H.A., West, J.D., Hotfilder, M., Heng, Y.M., Erickson, P., Calvo, R., Dalmau, J., Gemmill, R.M., and Sablitzky, F. (1999) DEF-3(g16/NY-LU-12), an RNA binding protein from the 3p21.3 homozygous deletion region in SCLC. *Oncogene* **18**, 2589–2597.
17. Edamatsu, H., Kaziro, Y., and Itoh, H. (2000) LUCA15, a putative tumour suppressor gene encoding an RNA-binding nuclear protein, is down-regulated in ras-transformed Rat-1 cells. *Genes Cells* **5**, 849–858.
18. Courey, A.J. and Tjian, R. (1988) Analysis of Sp1 in vivo reveals multiple transcriptional domains, including a novel glutamine-rich activation motif. *Cell* **55**, 887–898.
19. Gure, A.O., Altorki, N.K., Stockert, E., Scanlan, M.J., Old, L.J., and Chen, Y.T. (1998) Human lung cancer antigens recognized by autologous antibodies: definition of a novel cDNA derived from the tumor suppressor gene locus on chromosome 3p21.3. *Cancer Res.* **58**,1034–1041.
20. Hotfilder, M., Baxendale, S., Cross, M.A., and Sablitzky, F. (1999) Def-2, -3, -6 and -8, novel mouse genes differentially expressed in the haemopoietic system. *Br. J. Haematol*. **106**,335–344.
21. Scanlan, M.J., Gordan, J.D., Williamson, B., Stockert, E., Bander, H., Jongeneel, V., Gure, A.O., Jager, D., Jager, E., Knuth, A., Chen, Y.T., and Old, L.J. (1999) Antigens recognized by autologous antibody in patients with renal-cell carcinoma. *Int. J. Cancer* **83**, 456–464.
22. Sutherland, L.C., Edwards, S.E., Cable, H.C, Poirier, G.G., Miller, B.A., Cooper, C.S., and Williams, G.T. (2000) LUCA-15-encoded sequence variants regulate CD95-mediated apoptosis. *Oncogene* **19**, 3774–3781.
23. Yates, J.L., Warren, N., and Sudgen, B. (1985) Stable replication of plasmids derived from Epstein-Barr virus in various mammalian cells. *Nature* **313**, 812–815.
24. Mourtada-Maarabouni, M., Sutherland, L.C., Clark, J.P., Cooper, C.S., and Willimas, G.T. (2001) Regulation of T-cell apoptosis by sequences encoded at the LUCA-15 candidate tumour suppressor Locus. *Miami Nat. Biotechnol*. **12**, 40.
25. Mourtada-Maarabouni, M., Keen, J., Sutherland, L.S., Clark, J.P., Cooper, C.S., and Williams, G.T. (2002) The LUCA-15/RBM5 candidate tumor suppressor regulates T-cell apoptosis. *Am. Assoc. Cancer Res. Proc*. B-77.
26. Burd, C.G. and Dreyfuss, G. (1994) Conserved structures and diversity of functions of RNA-binding proteins. *Science* **265**, 615–621.
27. Clurman, B. and Groudine, M. (1997) Tumor suppressor genes. Killer in search of a motive? *Nature* **389**, 122–123.
